# Exploring over-the-counter SABA inhaler use in Malaysian community pharmacies: a mixed-methods study

**DOI:** 10.1080/20523211.2026.2750478

**Published:** 2026-11-06

**Authors:** Rabia Hussain, Zhe Chi Loh, Bayan Faisal Ababneh, Siew Chin Ong, Jaya Muneswarao

**Affiliations:** a School of Pharmaceutical Sciences, Universiti Sains Malaysia, Pulau Pinang, Malaysia; b Pharmacy Department, Hospital Pulau Pinang, Pulau Pinang, Malaysia

**Keywords:** Non-prescription medicines, asthma, over the counter, short-acting beta-2 agonists (SABA) inhalers, community pharmacists, community pharmacy

## Abstract

**Background:**

Non-prescription short-acting beta-2 agonists (SABA) inhalers, used to relieve asthma symptoms, are widely available in community pharmacies. This study aimed to explore the perceptions and practices of community pharmacists towards the safe use of SABA inhalers in Malaysia.

**Methods:**

A concurrent mixed-methods study was conducted from December 2022 to May 2023, involving qualitative interviews (n = 12) and quantitative online surveys (n = 312) among Malaysian community pharmacists. Qualitative data was managed through NVivo® software, and thematic analysis was performed. Descriptive data analysis, including Kruskal–Wallis and Mann–Whitney tests, and regression analysis were performed using IBM SPSS v.20 for the quantitative study. Subsequently, a comparison was made between the qualitative and quantitative data.

**Results:**

Most community pharmacists commonly linked effective asthma control with fewer exacerbations (n = 12) (84.3%), and decreased usage of SABA inhalers (n = 12) (65.4%), in the qualitative and quantitative studies, respectively. According to the quantitative results, 40.7% of participants were concerned with the frequency and dosage of SABA usage by asthma patients OTC. While in the qualitative phase, community pharmacists (n = 12) emphasised proper inhalation techniques for safe and effective use of non-prescription SABA inhalers. Community pharmacists from both studies suggested that effective counselling, including allocating sufficient time for counselling sessions, could enhance the safe use of SABA inhalers. The most agreed-upon perceived barriers to safe SABA inhaler use in the quantitative results were patients’ lack of awareness of available services for asthma consultations and counselling (74.4%) and insufficient time to provide counselling services (68.9%).

**Conclusion:**

Community pharmacists in Malaysia correlated good asthma control status with decreased exacerbations and SABA inhaler utilisation. Furthermore, community pharmacists were concerned about patients’ techniques, dosage, and frequency of use of SABA inhalers. However, asthma reviews, monitoring systems, and patient counselling were important suggested factors in ensuring safe and effective treatment in the community pharmacy setting.

## Background

Globally, around 300 million individuals (29%) suffer from asthma, including a significant portion of the Malaysian population (Dharmage et al., 2019; Global Initiative for Asthma, 2024). The asthma prevalence ranges from 8.9 to 13% in children (6–14 years) and 6.3% in adults in Malaysia (Ahad & Ming Khoo, 2017). Short-acting beta-2 agonists (SABA), relievers used as needed only, provide rapid relief of symptoms by relaxing bronchial smooth muscles and improving airflow (Global Initiative for Asthma, 2019). Non-prescription SABA inhalers, readily available at community pharmacies in several countries, including Australia, Russia, Italy, Egypt, Kenya, and Malaysia, offer convenient access to relief for patients suffering from asthma (Azzi et al., 2019; Loh et al., 2023a; Price et al., 2024a). As per the Global Initiative for Asthma (GINA) recommendation, even for mild cases of asthma, a low-dose inhaled corticosteroid (ICS)-formoterol is a preferred reliever option over SABA monotherapy for step one and two of asthma treatment guidelines (Global Initiative for Asthma, 2023). Alternatively, SABA can be utilised on an as-needed basis alongside ICS as an anti-inflammatory agent (Global Initiative for Asthma, 2023).

Nevertheless, instances of SABA overuse persist and raise the risk of hospitalisation and asthma flare-ups (Avdeev et al., 2022; Canonica et al., 2021). This overuse of asthma medication may arise from a reliance on bronchodilators (Global Initiative for Asthma, 2024). Moreover, the availability of non-prescription SABA inhalers at community pharmacies necessitates careful consideration due to the risk of inappropriate asthma management in the absence of medical supervision (Azzi et al., 2022, 2019; Price et al., 2024a). Asthma control is the extent of asthma features that can be observed in a patient, and it is assessed by symptom control and risk of adverse outcomes (Loh et al., 2023a). Patients with well-controlled asthma should not use SABA relievers more than twice per week (Loh et al., 2023a). Ensuring the safe use of SABA inhalers (appropriate dose, frequency, and techniques) may prevent adverse effects such as increased asthma exacerbations and hospitalisations (Kaplan et al., 2020). The collection of more than two SABA canisters within a one-year baseline period after inclusion was defined as SABA overuse (Nwaru et al., 2020). According to an Australian study, up to 66.4% of over-the-counter (OTC) SABA users were overusers (used more than two SABA canisters per year) (Global Initiative for Asthma, 2019; Price et al., 2024b).

Community pharmacists are positioned as accessible healthcare professionals, often serving as the first point for individuals seeking healthcare guidance or assistance (Loh et al., 2021). They play a crucial role in promoting healthcare services such as appropriate SABA use and asthma management (Ahmad et al., 2024; Buss et al., 2018; Hasan et al., 2021; Jailaini et al., 2024; Rasheed et al., 2020). Malaysia operates its primary care services within two parallel systems: publicly managed primary clinics regulated by the Ministry of Health and privately owned practices encompassing primary clinics and community pharmacies (Ong et al., 2022). Community pharmacists in Malaysia primarily dispense medications and dietary supplements, provide advice for minor medical issues, conduct health screenings, support smoking cessation endeavours, and facilitate weight management strategies (Hussain et al., 2025 ). According to Hamidi et al. (2021), 76.1% of Malaysians visited community pharmacies to obtain medicines and 5.0% consulted community pharmacists if they experienced any health concerns (Hamidi et al., 2021).

The rate of poorly controlled asthma cases remains elevated in Malaysia, imposing a significant burden on healthcare resources and expenditure (Hussein et al., 2023). In Malaysia, SABA inhalers or tablets can be purchased from community pharmacists without a prescription, which accounts for a large proportion of the total number of SABAs dispensed annually (Ban et al., 2023).

According to a study conducted in Malaysian health clinics, 29.4% of adults and 13.8% of children had uncontrolled asthma (Hussein et al., 2023). Notably, the urgent care costs for asthma patients surpassed maintenance costs in Malaysia, highlighting the need for proactive asthma management approaches to mitigate healthcare expenses over time (Bahadori et al., 2009). As per findings of a Malaysian study, the mean (± SD) cost of asthma maintenance management was $48.04. The cost of acute exacerbation management in the emergency department was $13.50 and in the medical ward, the cost was $552.13 per hospitalisation event (Yong & Shafie, 2018). Also, low- to middle-income countries faced challenges related to the quality of practice encompassing dispensing, labelling, and counselling, and issues with the consistent supply of medications (Hamid et al., 2020; Smith, 2009).

Studies show that community pharmacists perceived that patients were not concerned about asthma, and the use of asthma relievers has been normalised in the community (Alyas et al., 2024; Bereznicki, Peterson, Jackson, Haydn Walters, et al., 2011; Bereznicki, Peterson, Jackson, Walters, et al., 2011; Bereznicki et al., 2013). However, data regarding community pharmacists’ perceptions and practices towards the use of SABA inhalers were limited. Therefore, understanding community pharmacists’ perspectives regarding the use of SABA inhalers is essential in Malaysia.

## Objective

The research aimed to explore community pharmacists’ perceptions and practices towards the safe use of non-prescription SABA inhalers at community pharmacies in Malaysia.

## Methods

### Study design

This study utilised a mixed-methods design comprising a concurrent approach, meaning that both qualitative and quantitative data were collected simultaneously, analysed separately, and interpreted together (Creswell et al., 2018). The quantitative and qualitative findings enabled a comparative analysis, enhancing the data credibility through convergence and divergence interpretations (Plano Clark et al. 2015; Pluye et al., 2009, 2018). Divergence, which was defined as conflict, contradiction, discordance, discrepancy, dissonance, and inconsistency between qualitative and quantitative data and findings, leading to the absence of harmony between data and results (Morgan, 2019). Conversely, convergence was referred to as the harmony and consistency of quantitative and qualitative data and results (Pluye et al., 2009, p. 2018).

The exploratory qualitative study was conducted through semi-structured interviews and the quantitative cross-sectional study through online surveys among community pharmacists in Malaysia. The phenomenology approach was followed for the qualitative study to explore the participants’ views and experiences (Tariq, 2013). The Good Reporting of a Mixed Methods Study (GRAMMS) guidelines were followed for the reporting of methodology (Supplemental Material Appendix A).

### Participants and sample size

This study was conducted at community pharmacies in Malaysia from December 2022 to May 2023. Participants were recruited through purposive and snowball sampling for the qualitative study, and for the quantitative survey, the target population was recruited through purposive and convenience sampling approach (Smith, 2010; Tongco, 2007). Eligible participants for both qualitative and quantitative studies were community pharmacists, registered with the Pharmacy Board of Malaysia, who held full-time permanent positions within community pharmacies, were proficient in English, and had provided informed consent. Part-time pharmacists, assistant pharmacists, and trainees were excluded. The recruitment process of community pharmacists for interviews is shown in Figure 1, while the survey was transformed into a Google Form and disseminated through various professional pharmacist groups on platforms like Facebook, WhatsApp, Telegram, and emails, leveraging the benefits of convenience, expanded reach, cost-effectiveness, and the option for respondents to remain anonymous (Vasantha Raju & Harinarayana, 2016). Data collection for the qualitative study was conducted until data saturation was achieved, in which no new information or themes were discovered (Hennink & Kaiser, 2022; Saunders et al., 2018). A sample of 12 community pharmacists was interviewed in the qualitative study. While data saturation was achieved after 10 pharmacists’ interviews, a further two interviews were carried out to verify the saturation of the data.

**Figure 1.**
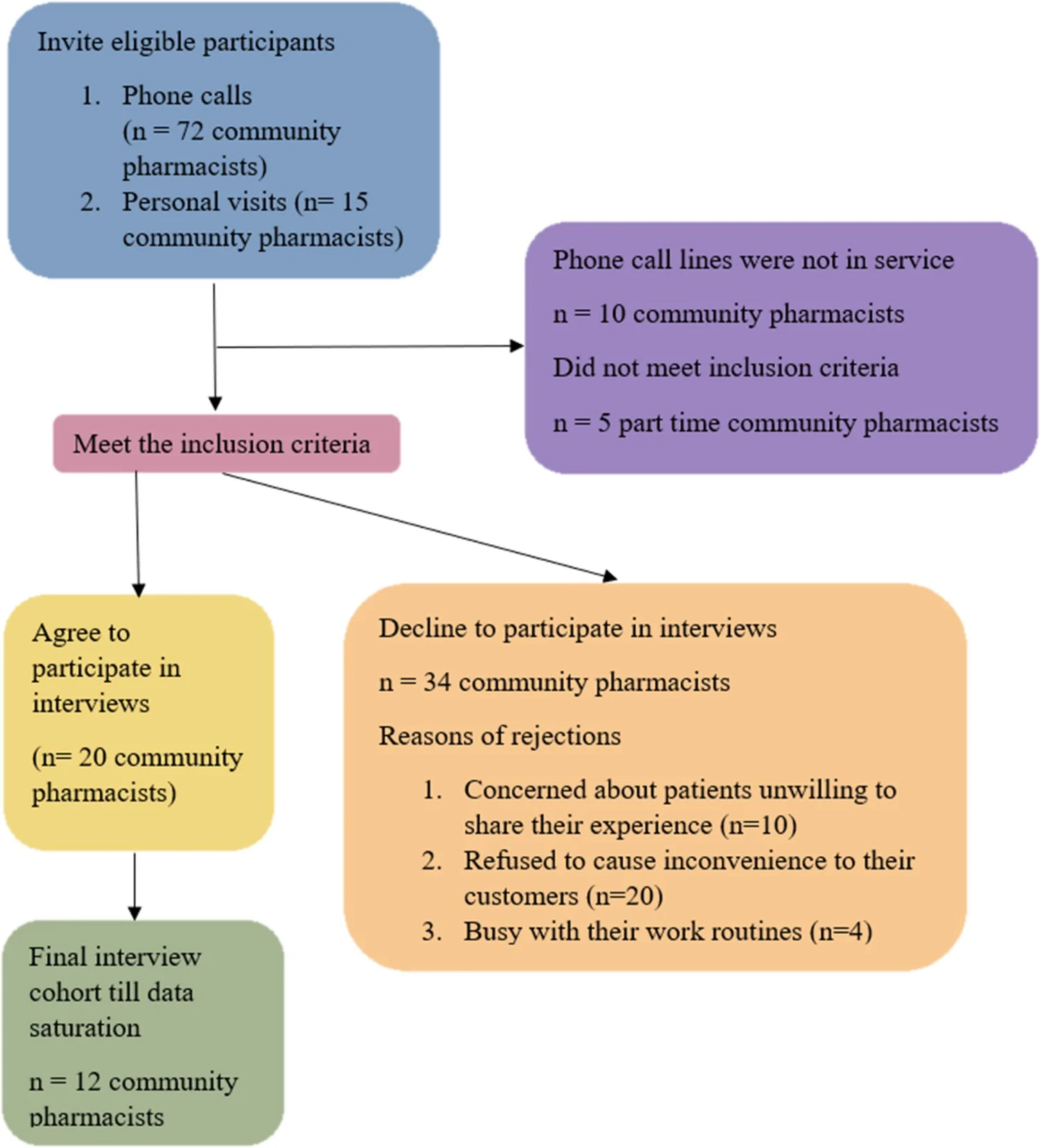
The recruitment process for the qualitative study.

The sample size of the quantitative study was 312 community pharmacists, and it was calculated by using the Cochrane formula (Nundy et al., 2022).

## Data collection

### Qualitative study

A semi-structured interview guide was developed (Supplemental Material Appendix B) following a thorough literature review (Azzi et al., 2022, 2019; Blakeston et al., 2021; Loh et al., 2023a; Muneswarao et al., 2019) and considered the opinions of experts in the field. It was then reviewed by academics and pilot-tested with two community pharmacists. The guide was supplemented with probes and prompts and focused on asthma control and management, risk perceptions of the use of SABA inhalers, and communication about the safe use of SABA inhalers.

The interviewer (ZCL), previously trained in qualitative interviews, conducted the interviews in person at community pharmacies or through the Zoom® application (version 5.14.11) at a date and time convenient for the participants. Before the interviews, the interviewer briefed participants about the research objectives. Moreover, participants were free to express their views openly during the 30–40 -minute interviews without any influence from leading questions. The interviewer took some field notes during the interviews to aid in data interpretation. Data were recorded and saved securely on a password-protected computer. The findings of the qualitative study were reported using the Consolidated Criteria for Reporting Qualitative Studies (COREQ) checklist (Fusch et al., 2015; Hussain et al., 2018; Tongco, 2007; Vasileiou et al., 2018).

Quantitative study. After an extensive literature review (Azzi et al., 2019, 2022; Blakeston et al., 2021; Chan et al., 2020; Kaplan et al., 2020; Loh et al., 2023a, 2023b), a closed-ended questionnaire (Supplemental Material Appendix C) was designed to evaluate the perceptions and practices of community pharmacists regarding the safe use of non-prescription SABA inhalers. The final questionnaire comprised of the following six sections:

Section 1: sociodemographic characteristics of participants, Section 2: perceptions of asthma control and OTC SABA inhaler use, Section 3: risk perceptions of asthma reliever inhalers, Section 4: dispensing and education practices for safe use of OTC SABA inhalers, Section 5: facilitators and barriers to the safe use of SABA inhalers.

A five-point Likert scale (‘strongly disagree', ‘disagree', ‘neutral', ‘agree', and ‘strongly agree') was employed to rate all the questions.

### Validity and reliability

Qualitative study: To enhance the applicability, credibility, consistency, and confirmability of the qualitative study, several approaches were taken. For example, the study team had access to digital recordings to ensure the accuracy of data transcription. There was no collaboration between the researchers and the subjects in this study. Besides the primary researcher (interviewer), two more researchers with expertise in qualitative research methods supervised the entire study. Furthermore, another researcher, who specialised in qualitative research techniques, conducted a thematic analysis and validated the themes. To ensure the researchers’ reflexivity, some participants’ interview transcripts were returned to them via WhatsApp, and they were satisfied with the transcribed words.

Quantitative study: Four academics and subject-matter specialists comprised the evaluation team who examined the questionnaire’s content validity. A content validity form was created with a five-point Likert scale and sent to the experts via email. The experts were asked to assess whether all items referred to the pertinent aspects of the constructs to be measured (1 = relevant, 5 = irrelevant), the significance of each item (1 = essential, 5 = not necessary), and any items that were missing from the questionnaire. To assess the questionnaire’s face validity and reliability, pilot testing was employed among 40 community pharmacists who were excluded from the final analysis. Participants offered feedback on the items’ level of clarity (1 = the statement is very vague, 5 = the sentence is very clear) and comprehension (1 = the sentence is difficult to understand, 5 = the sentence is easy to understand) through a five-point Likert scale concerning face validity. Reliability was tested via Cronbach's alpha test, yielding a coefficient of 0.731 for the total scale, which indicated acceptable reproducibility and consistency of the questionnaire (Cronbach, 1951). Two new items were added to the original 25-item questionnaire in response to expert input. After this feedback, five items were further clarified.

### Data analysis

Qualitative study: The qualitative data analysis employed an inductive approach and adhered to the techniques described by Braun and Clarke (2006) (Braun & Clarke, 2006; Hussain et al., 2018). Thematic analysis was started by researchers familiarising themselves with the transcript data. Through a constant-comparative method, line-by-line coding was performed independently by two researchers (ZCL and RH) using NVivo software®. Different opinions in the coding process were resolved through discussion. Potential themes were developed by grouping codes that were related to each other. Codes and themes were reviewed and refined continuously through discussions (Broom, 2005; Ezzy, 2002). Spot-checking of coding and thematic analysis was also conducted by two team members (SCO, BA) to ensure the quality of data analysis.

Quantitative study: In terms of quantitative data analysis, data analysis was conducted using IBM SPSS® v.20. Descriptive analyses were utilised to present the data in frequencies and percentages. Additionally, the Kruskal–Wallis test and Mann–Whitney U test were employed for inferential analysis due to the non-normal distribution of data. Regression analysis was carried out to identify associated predictors of sociodemographic characteristics of the participants regarding perception of asthma control and management. A *p*-value less than 0.05 was considered statistically significant.

Subsequently, a comparison was made between the qualitative and quantitative data. For that comparison, the five-point Likert scale was summarised and presented as a three-point Likert scale: ‘disagree', ‘neutral', and ‘agree'.

## Ethical approval and informed consent

The Human Research Ethics Committee, namely Jawatankuasa Etika Penyelidikan Manusia of Universiti Sains Malaysia (USM), has approved this study under the reference number USM/JEPeM/22090575. Informed consent was obtained from all the participants who participated in both the qualitative and quantitative studies. The identity of the participants who participated in the qualitative study was kept confidential to ensure complete privacy with researchers by assigning codes to each participant. Moreover, responses from the quantitative study were anonymised. Participation in the study was completely voluntary; no incentives were offered, and participants had the right to withdraw from this study at any point without providing any reason.

## Results

### Demographics of the participants

A total of 12 and 312 participants were included in the qualitative and quantitative analyses. The majority of the participants in both studies were aged between 29–38 years old, female, Chinese, and had a bachelor’s degree. The details of participants’ demographics in both studies are described in Table 1.

**Table 1.** Demographics of community pharmacists.

Characteristics	Frequency (n)	
	Qualitative study (N = 12)	Quantitative study(N = 312)
Age (years)		
18–28	2	93
29–38	7	159
39–48	1	29
49 years old and above	2	31
Gender		
Male	3	112
Female	9	200
Ethnicity		
Malay	1	95
Chinese	9	188
Indian	2	29
Education level		
Bachelor’s degree	11	298
Master’s degree	1	13
Years in practice		
Less than 5 years	3	110
5–10 years	4	140
10 years and above	5	62
States of residence		
Penang	7	80
Perak	5	17
Perlis	-	5
Sabah	-	9
Sarawak	-	13
Selangor	-	64
Terengganu	-	11
Kuala Lumpur	-	15
Labuan	-	1
Johor	-	41
Kedah	-	30
Kelantan	-	7
Melaka	-	12
Pahang	-	6

An overall summary of comparisons between qualitative and quantitative study findings has been described in Table 2.

**Table 2.** Comparisons between qualitative and quantitative studies.

Qualitative quotes	Summary	Quantitative results	Summary	Meta inferences
Perceptions and understandings about good asthma control				
*‘Patients with good asthma control are those who have asthma attacks (symptoms) less than three times a week.'* (Female, Pharmacist-02) ‘*I consider a patient to have good asthma control if they have fewer asthma attacks (symptoms), perhaps less than five in a month. In that case, their asthma can be considered well-controlled'.* (Female, Pharmacist-08)	All community pharmacists (n = 12) identified individuals with effective asthma control as those who encountered infrequent asthma exacerbations and reduced SABA usage.	According to you, good asthma control is: (a) Asthma that can be controlled with medical help. (b) Having minimum asthma symptoms. 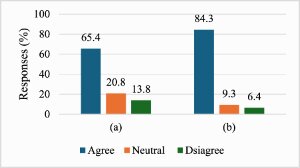	Most community pharmacists agreed that good asthma control is asthma that can be controlled with medical help (65.4%) and having minimum asthma symptoms (84.3%).	A convergence finding highlighted that effective asthma control involved minimising asthma exacerbations and reducing the usage of SABA inhalers.
Asthma reviews				
*‘Maybe patients’ asthma status was not diagnosed accurately. Their asthma condition might worsen, and they need some add-on therapy, but the patients themselves did not realise this and nobody told them about this, so they just keep using the SABA inhalers. We have to ask them to go back to their doctors and follow their doctor's prescriptions’.* (Female, Pharmacist-08) ‘*Their symptoms are not really assessed, so they might overuse it, but then they are not sure when to go and see a doctor. And then some of the patients are like, they refused to see a doctor no matter what their conditions are’.* (Male, Pharmacist-12)	Most community pharmacists (n = 5) identified that frequent SABA users needed to be more aware of the necessity of asthma follow-ups to monitor their current asthma status.	Asthma patients who always purchase SABA inhalers from community pharmacies will have poor asthma control due to absence of asthma reviews at the hospital. 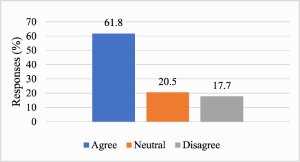	61.8% of participants perceived that asthma patients who always purchased SABA inhalers at community pharmacies will have poor asthma control due to the absence of asthma reviews.	From community pharmacists’ perceptions, SABA users need to have regular asthma reassessments.
Reliance on SABA inhalers				
*‘SABA inhalers can provide immediate relief for their symptoms, unlike budesonide beclomethasone, which doesn't offer immediate effects. They don't notice the impact. Therefore, we need to continue counselling them and encourage reduced reliance on SABA inhalers’.* (Female, Pharmacist-05) ‘*From what I observe in the community, patients are unaware that SABA is solely for short-term relief. Once they experience the comforting sensation upon inhalation, they become attached to what brings them comfort’.* (Male, Pharmacist-07)	More than half of the community pharmacists (n = 7) raised concerns regarding asthma patients who tend to depend on the use of SABA inhalers for immediate relief effects.	I think asthma patients develop attachment to SABA inhalers. 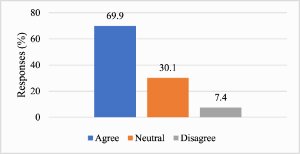	69.9% of participants perceived that asthma patients tend to develop a dependence on SABA inhalers.	Community pharmacists generally agreed that asthma patients developed reliance on SABA inhalers.
Practices towards the proper use of SABA inhalers				
*‘The frequency of their usage is what I care about the most. The second concern is dosage, and techniques come last. In terms of techniques, patients have made significant improvements. They all know how to use it now'.* (Female, Pharmacist-04*)* ‘*My main concerns are the frequency and dosage of SABA inhaler use because excessive usage can lead to poor asthma control'.* (Female, Pharmacist-09)	All community pharmacists expressed particular concern regarding the correct dosage, frequency, and technique employed by asthma patients when using SABA inhalers.	I am more concerned about the frequencies and doses of asthma patients’ SABA usage compared with the techniques to utilise the inhalers. 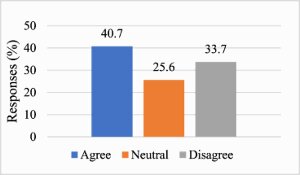	Interestingly, 40.7% of participants were more concerned about the frequencies and dosage of asthma patients’ SABA usage compared to their techniques to use inhalers.	Community pharmacists in the qualitative study were more concerned about the techniques to use SABA inhalers, while those included in quantitative study were more focused on frequency and dosage.
A monitoring system for non-prescription SABA inhalers				
*‘Patients can purchase SABA inhalers over-the-counter from nearby pharmacies at any time, contributing to their ease of access’.* (Female, Pharmacist-09*)* ‘*They don't really follow up with their doctors; they simply come and purchase the medication. They use it quite frequently’.* (Female, Pharmacist-03*)* ‘*Within hospital or Klinik Kesihatan (government health clinic) settings, patients are closely monitored for their SABA usage. The government provides a one-to-one exchange system for inhalers, where patients must return their empty canister to receive a new one. Pharmacists keep records on appointment cards, and if they notice frequent visits, they take action. However, in the community setting, there is limited monitoring due to the ease of obtaining SABA inhalers from retail pharmacies for around RM 20 (USD 4)’.* (Female, Pharmacist-10)	Community pharmacists perceived that patients prefer to purchase SABA inhalers at community pharmacies.	Perceived facilitator to the safe use of SABA inhalers: A monitoring system to keep track of the SABA inhalers purchasing records in the community setting. 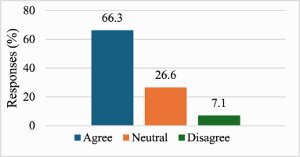	66.3% of participants agreed that a monitoring system should exist to keep track of the SABA purchasing records in the community pharmacies.	Community pharmacists identified asthma patients preferred to purchase SABA inhalers from community pharmacies, and the availability of a monitoring system could increase the safe use of SABA inhalers.
Asthma counselling				
*‘I believe it comes from communication errors, where misunderstandings can occur when Malay patients interact with Chinese healthcare professionals, or vice versa. Both parties may struggle to fully understand each other due to dialect and language differences during counselling sessions. Patients may simply say “yes” to what the healthcare provider says without fully understanding the message. This “yes” response might lead the healthcare provider to assume complete comprehension’.* (Male, Pharmacist-07) ‘*I think government hospitals should provide detailed and extended counselling sessions for asthma patients’.* (Female, Pharmacist-08)	Community pharmacists (n = 3) noted that language barriers in healthcare settings can hinder effective communication and message delivery.	Facilitator to the safe use of SABA inhalers: improve the duration of asthma counselling. 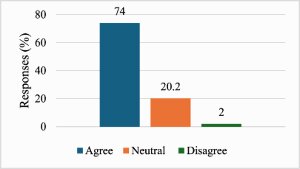	74% of community pharmacists participants identified improved duration of asthma counselling as facilitator to safe use of SABA inhalers and effective asthma management.	Regarding asthma counselling, community pharmacists in the qualitative study suggested to reduce language barriers, while community pharmacists in the quantitative study suggested to improve the duration of asthma counselling.
Foster public education / training				
*‘I think the Ministry of Health could do several things. To ensure that pharmacists have the skills necessary to work well in the counselling section, the training should first be made compulsories for all pharmacists, and they have to at the very least pass the exams.'* (Male, Pharmacist-07) ‘*Government must periodically offer training courses and invite community pharmacists to participate. They may learn how to communicate with asthma patients more effectively at one of these training sections. The pharmacists should be trained to improve their listening skills, as well as introduction to strategies like “teach-back” or “show-back” method'.* (Female, Pharmacist-09)	Half of the community pharmacists (n = 6) suggested establishing training programmes for community pharmacists regularly.	Barrier to the safe use of SABA inhalers: lack of training 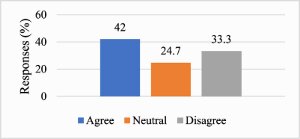	42% of participants agreed that lack of training is one of the barriers to the safe use of SABA inhalers.	Most participants agreed that lack of education and training is one of the barriers to the safe use of SABA inhalers.

## Perception and understanding of good asthma control

During the qualitative phase of the study, all community pharmacists were aware of good asthma control, as they agreed that good asthma control is related to reduced SABA usage and having minimal asthma symptoms, as exemplified below:

Patients with good asthma control are those who have asthma attacks (symptoms) less than three times a week. (Female, Pharmacist-02)

I consider a patient to have good asthma control if they have fewer asthma attacks (symptoms), perhaps less than five in a month. In that case, their asthma can be considered well-controlled. (Female, Pharmacist-08)

The subsequent quantitative findings further supported that, with the majority of community pharmacists perceiving that achieving good asthma control entailed managing the condition with medical assistance (65.4%) and experiencing minimal asthma symptoms (84.3%).

## Perceptions and experiences towards asthma reviews:

Most community pharmacists (n = 5) identified that frequent SABA users needed to be more aware of the necessity of asthma follow-ups to monitor their current asthma status, and they may need to receive additional treatments, such as inhaled corticosteroids:

Maybe patients’ asthma status was not diagnosed accurately. Their asthma condition might worsen, and they need some add-on therapy, but the patients themselves did not realize this, and nobody told them about this, so they just keep using the SABA inhalers. We have to ask them to go back to their doctors and follow their doctor's prescriptions. (Female, Pharmacist-08)

Community pharmacists encouraged patients to get a thorough asthma review with their physicians. After a thorough checkup, physicians may prescribe inhaled corticosteroids as a long-term treatment option. Community pharmacists claimed that:

I encourage them to undergo a comprehensive asthma review with physicians, as there may be underlying issues with their asthma. (Female, Pharmacist-08)

Similarly, in the quantitative survey, concerns were raised by most of the participants (61.8%, n = 193) that patients may not attend asthma reviews at the hospital and purchase non-prescription SABA inhalers at the community pharmacies.

## Perceptions about reliance and the over-the-counter availability of SABA inhalers:

More than half of the community pharmacists (n = 7) raised concerns regarding asthma patients who tend to depend on the use of SABA inhalers for immediate relief effects. According to them, community pharmacists must monitor and counsel patients properly, as overuse of SABA inhalers will increase asthma exacerbations.

I believe most asthma patients are unaware that SABA is a reliever, which lacks anti-inflammatory effects. They depend on it because it effectively alleviates their symptoms. They are unaware that frequent use of SABA inhalers can lead to an increase in asthma exacerbations. (Female, Pharmacist-09)

Community pharmacists perceived that patients prefer to purchase SABA inhalers at community pharmacies. One of the pharmacist participants mentioned that there was a lack of a monitoring system to trace SABA uses in community pharmacy settings. However, patients were monitored in the government setting because patients must return their empty canisters to receive a new one in Malaysia.

Patients can purchase SABA inhalers over-the-counter from nearby pharmacies at any time, contributing to their ease of access. (Female, Pharmacist-09)

Within hospital or Klinik Kesihatan (government health clinic) settings, patients are closely monitored for their SABA usage. The government provides a one-to-one exchange system for inhalers, where patients must return their empty canister to receive a new one. Pharmacists keep records on appointment cards, and if they notice frequent visits, they take action. However, in the community setting, there is limited monitoring due to the ease of obtaining SABA inhalers from retail pharmacies for around RM 20 (USD 4). (Female, Pharmacist-10)

The quantitative survey findings also illustrated that 68.6% of the online survey respondents agreed that asthma patients were highly likely to overuse non-prescription SABA inhalers obtained from community pharmacies compared to those obtained from hospitals or clinics. Moreover, 69.9% of participants perceived that patients tend to develop a dependence on SABA inhalers.

## Practices towards the proper use of SABA inhalers and the provision of pharmacists in SABA use assessment

All community pharmacists expressed particular concern regarding the correct dosage, frequency, and technique employed by patients when using SABA inhalers. Most of the community pharmacists (n = 7) mentioned that they always enquire about their patients’ frequency of SABA use. Some of them also investigated the potential reasons for SABA overuse.

The frequency of their usage is what I care about the most. The second concern is dosage, and techniques come last. In terms of techniques, patients have made significant improvements. They all know how to use it now. (Female, Pharmacist-04)

My main concerns are the frequency and dosage of SABA inhaler use because excessive usage can lead to poor asthma control. (Female, Pharmacist-09)

I've noticed that most patients are using their inhalers incorrectly. When they don't use the correct techniques, it's useless for them to use SABA inhalers because they can't effectively control their asthma. (Female, Pharmacist-06)

Initially, we will enquire about the frequency of inhaler use. Additionally, I will help them recognize potential triggers such as air pollution, allergens from pets’ fur, and so on. (Male, Pharmacist-07)

The subsequent quantitative phase findings also revealed that 40.7% of participants were more concerned about the frequencies and dosage of asthma patients’ SABA usage compared to their techniques for using inhalers. Additionally, most community pharmacists (93.5%) stated that they had educated patients on proper inhaler techniques, and 79.5% believed they provided adequate information to asthma patients. Additionally, most community pharmacists (93.5%) stated that they had educated patients on proper inhaler techniques, and 79.5% believed they provided adequate information to asthma patients.

## Barriers to the safe use of SABA inhaler

Three community pharmacists noted that language barriers in healthcare settings can hinder effective communication and message delivery.

At times, they face challenges communicating with their doctors, especially those in government hospitals where there may be language differences, particularly for Chinese or Indian patients who are less familiar with the Malay language. (Female, Pharmacist-05)

I believe it comes from communication errors, where misunderstandings can occur when Malay patients interact with Chinese healthcare professionals, or vice versa. Both parties may struggle to fully understand each other due to dialect and language differences during counselling sessions. Patients may simply say ‘yes’ to what the healthcare provider says without fully understanding the message. This ‘yes’ response might lead the healthcare provider to assume complete comprehension. (Male, Pharmacist-07)

Some community pharmacists, especially females (n = 5), who were involved in counselling sessions, urged that healthcare professionals should be more attentive to patients with low literacy levels and use simple language.

I believe that individual counselling could enhance communication between patients and pharmacists. Written instructions alone are not effective, as patients may struggle to understand the intended message. (Female, Pharmacist-02)

Another thing is about the elderly and children. In this case, we will ask the caretakers to come along and counsel them together. So that at least there is someone to take care of this and beware of this, to avoid misuses. Then we to use simple language to explain to them, avoid those medical words. (Female, Pharmacist-10)

Furthermore, the quantitative phase also complemented the qualitative findings, whereby the most agreed-upon perceived barriers to safe SABA inhaler use were patients’ lack of awareness of available services for asthma consultations and counselling (74.4%) and insufficient time to provide counselling services (68.9%).

## Facilitators to the safe use of SABA inhaler

Reducing the waiting time of patients and fostering public education were perceived as facilitators to the safe use of the SABA inhaler by community pharmacists during qualitative interviews. Two Chinese community pharmacists claimed that their job routine was very busy and they seldom had time for detailed counselling of patients.

Considering everyone's busy schedules, most patients simply come to obtain their prescriptions and have limited time for extended interactions. (Female, Pharmacist-09)

If there are no customers, perhaps we can allocate more time for counselling. However, we often find ourselves busy, resulting in shortened counselling sessions. (Female, Pharmacist-11)

Half of the community pharmacists recommended establishing training programmes for community pharmacists regularly. It would improve their communication skills to introduce techniques such as the ‘teach back’ or ‘show back’ method.

I think the Ministry of Health could do several things. To ensure that pharmacists have the skills necessary to work well in the counselling section, the training should first be made compulsories for all pharmacists, and they have to at the very least pass the exams. (Male, Pharmacist-07)

I think government hospitals should provide detailed and extended counselling sessions for asthma patients. (Female, Pharmacist-08)

Government must periodically offer training courses and invite community pharmacists to participate. They may learn how to communicate with asthma patients more effectively at one of these training sections. The pharmacists should be trained to improve their listening skills, as well as introduction to strategies like ‘teach-back’ or ‘show-back’ method. (Female, Pharmacist-09)

These findings were further supported by the quantitative results, as offering more follow-up sessions for asthma patients (75.4%) and extending the duration of asthma counselling (74.0%) were reported as facilitators in the survey that could enhance the safe use of SABA inhalers.

## Discussion

This was a concurrent mixed-methods study to explore the perceptions and practices of community pharmacists towards the safe use of SABA inhalers at community pharmacies in Malaysia. This mixed-methods study has compared the qualitative and quantitative data, providing a more holistic and robust comprehension of the subject matter (Skamagki et al., 2024).

Community pharmacists in both qualitative and quantitative studies commonly linked effective asthma control with fewer exacerbations and decreased usage of SABA inhalers. This perception aligns with research that defines asthma control in terms of symptom management and minimising the risk of future exacerbations (Global Initiative for Asthma, 2021). Moreover, well-managed asthma is characterised by reduced reliance on SABA inhalers and healthcare services (Menzies-Gow et al., 2022). The rate of poorly controlled asthma cases remains elevated in Malaysia, imposing a significant burden on healthcare resources and expenditure (Hussein et al., 2023). In this study, community pharmacists perceived that overuse of SABA inhalers related to the patient’s lack of asthma reviews with physicians. It has been suggested in the literature that proactive communication during follow-up sessions is considered important to ensure continuous monitoring and adjustment of treatment plans (Carlisle et al., 2011; Price et al., 2016). A systematic review indicated that regular monitoring and follow-up with asthma patients could reduce the overuse of OTC SABA inhalers (Loh et al., 2023b). The easy accessibility of non-prescription SABA inhalers at community pharmacies may pose both beneficial and adverse outcomes. While it could benefit patients by offering convenience, it may also lead to poor asthma control if SABA inhalers are not used safely due to inappropriate inhalation techniques, dosages, and frequency (Loh et al., 2023a). It has been reported that 70.1–73.9% of asthma patients who purchased non-prescription SABA at community pharmacies were SABA overusers (Azzi et al., 2019, 2022; Loh et al., 2023a). To mitigate this issue, community pharmacists, being highly accessible, are well-positioned to educate patients on asthma and SABA inhaler use, with evidence from countries like the UK, Australia, Canada, and Germany showing improvements in asthma control, quality of life, and medication adherence (Brown et al., 2017; Jarab et al., 2024; Mahdavi & Esmaily, 2021). Additionally, a monitoring and surveillance system for the frequency of non-prescription SABA purchases should be implemented to reduce cases of SABA overuse (Cooper, 2013; Loh et al., 2023b).

As reported in our studies, community pharmacists expressed concerns about asthma patients’ tendency to over-rely on SABA inhalers for their immediate relief effects. This reliance often arises from the rapid symptom alleviation provided by these inhalers (Blakeston et al., 2021; Loh et al., 2023a). Over time, some patients may develop an emotional attachment to SABA inhalers, particularly those who have been using them since their childhood (Blakeston et al., 2021; Loh et al., 2023a). Healthcare professionals can utilise various tools to effectively identify patients who rely on SABA inhalers and prioritise interventions accordingly (Ban et al., 2024). Examples of such tools include the Reliever Reliance Test (RRT) and the SABA Reliance Questionnaire (SRQ) (Ban et al., 2024).

The quantitative results revealed that 40.7% of participants were primarily concerned with the frequency and dosage of asthma patients who were OTC using SABA. In contrast, the qualitative findings emphasised proper inhalation techniques to ensure the safe and effective use of non-prescription SABA inhalers. Overuse of SABA inhalers can lead to an increase in side effects such as tremors, palpitations, and hypokalaemia, as well as it could mask underlying poor asthma control (Garzon-Siatoya et al., 2022). Optimal inhaler techniques can achieve the desired pharmacological effects, preventing patients from overusing SABA inhalers to control their exacerbated symptoms (Shahid et al., 2022).

The study also reported that community pharmacists perceived that patients prefer to purchase SABA inhalers at community pharmacies. Community pharmacists mentioned that they always enquire about their patients’ frequency of SABA use, and some of them also investigated the potential reason for SABA overuse. Controlling the use of SABA inhalers in Malaysia has been challenging, as they can be obtained from various sources, including public health clinics and community pharmacies (Ban et al., 2024). Monitoring daily SABA use helps prevent asthma exacerbations by prompting patients to follow their asthma action plan (Amirav et al., 2023). Additionally, community pharmacists should control the dispensing of SABA inhalers (Ban et al., 2024). To address this issue, the Global Policy Steering Group on Asthma recommends that SABAs should only be provided to patients with a valid prescription or in a clinical emergency (e.g. exacerbation), and that SABA should not be dispensed as a standalone treatment (Kaplan et al., 2021).

Asthma education programmes should prioritise proper SABA usage, with trained pharmacists explaining its proper frequency and techniques (Hussain & Paravattil, 2020). Asthma education could be more effective if patients are willing to engage with community pharmacists, leading to better asthma control and higher expectations for treatment outcomes (Qazi et al., 2021).

Community pharmacists identified language barriers in healthcare as key hindrances to communication and effective SABA inhaler counselling. Particularly for patients with limited health literacy and who do not understand the doctor’s spoken language. Additionally, patients’ unawareness of services and time constraints were also reported as barriers to the safe use of SABA. Similar barriers were reported by a study conducted among community pharmacists in Saudi Arabia dealing with asthma patients (Alzayer, 2023). According to the study, the language barriers caused miscommunication with patients because pharmacists faced some challenges dealing with Saudi patients due to different accents (Alzayer, 2023). Moreover, a shortage of time during working hours prevents them from teaching patients about medications (Alzayer, 2023).

Community pharmacists from both studies suggested that effective counselling, including the allocation of sufficient time for counselling sessions, could enhance the safe use of SABA inhalers. This corroborates with the literature about pharmacists’ effective communication skills that include recognising the psychological background of their patients and understanding patients’ opinions regarding their conditions (Othman et al., 2023). Effective counselling strategies involve avoiding the use of medical jargon and instead utilising simple language and words appropriate for the literacy level of patients (Delli et al., 2022; Munsour et al., 2025). Introduction of the Teach-Back method can also help in the safe use of SABA. The Teach-Back method entails patients repeating health information to ensure comprehension (Shersher et al., 2021).

A study from Belgium suggested that patients with difficult-to-control asthma, who were under long-term ICS therapy, had a pharmacy care (PC) intervention involving counselling from community pharmacists (Putman et al., 2022). This intervention notably increased ICS usage while reducing the use of SABA inhalers (Putman et al., 2022). Additionally, healthcare professionals have suggested that providing asthma education, a written asthma action plan, and educating patients about inhaler use techniques at the time of their discharge from hospitals can help patients effectively adhere to their treatment options (Ban et al., 2024).

## Strengths and limitations

The quantitative and qualitative findings enabled a comparative analysis, enhancing the data credibility through convergence and divergence interpretations. Although the qualitative sample was modest and limited in its nationwide generalisability, the results remain valuable for healthcare professionals as they are complemented by a quantitative study, which provides a statistical perspective on the issue. The qualitative segment involved participants from two states (Penang and Perak), while the quantitative component encompassed participants from across the nation. Despite potential biases from self-reporting, such as social desirability and recall bias, these were minimised by ensuring participants’ anonymity using a validated interview guide and shortening the recall period. Additionally, the questionnaire was distributed online, which targeted individuals with internet access, and responses from areas lacking such accessibility may not be captured. This may lead to demographic selection bias. However, this study marks the first instance of employing a concurrent mixed methods approach to explore the perceptions and practices of community pharmacists regarding the safe utilisation of SABA inhalers.

## Conclusion

In conclusion, community pharmacists in Malaysia linked good asthma control status with decreased exacerbations and SABA inhaler utilisation. Furthermore, community pharmacists in both qualitative and quantitative studies were concerned about patients’ techniques, dosage, and frequency of SABA inhaler use. Asthma reviews, monitoring systems, and patient counselling were important suggested factors to ensure safe and effective treatment in community pharmacy settings in both studies. Future studies are needed to explore the perceptions of other healthcare professionals towards the use of SABA inhalers in different healthcare settings in Malaysia.

## Supplementary Material

Appendices.docx

## Availability of materials and data

All data generated or analysed during this study are included in this published article (and its Supplemental Material files).
