# Supplementary material for: Exploring over-the-counter SABA inhaler use in Malaysian community pharmacies: a mixed-methods study
Source: J Pharm Policy Pract. 2026 Nov 6;19(1):2750478. doi: 10.1080/20523211.2026.2750478 (PMC13757800; doi:10.1080/20523211.2026.2750478)
Supplement: Supplementary file 1 [file JPPP_A_2750478_SM6306.docx]

**Appendix A**

**Good Reporting of A Mixed Methods Study (GRAMMS) guidelines**

| **Guidelines** | **Page** |
| --- | --- |
| 1. Describe the justification for using a mixed methods approach to the research question. | 3 |
| 2. Describe the design in terms of the purpose, priority and sequence of methods. | 4-8 |
| 3. Describe each method in terms of sampling, data collection and analysis. | 4-8 |
| 4. Describe where integration has occurred, how it has occurred and who has participated in it. | 4-8 |
| 5. Describe any limitation of one method associated with the present of the other method. | 15 |
| 6. Describe any insights gained from mixing or integrating methods | 8-15 |

**Appendix B**

**Community pharmacists’ interview guide**

Part I

Focus: Asthma control and management

1. How do you describe patients with good asthma control?
2. Would you dispense the reliever inhalers to asthma patients if you noticed they might overuse it? Could you please provide the reasons?

Part II

Focus: Risk perceptions towards the use of SABA inhalers

1. Are you concerned about safe use of SABA inhalers by asthma patients?
2. In your opinion, what could be the reasons for patients overusing SABA inhalers which are requested from community pharmacies?

Part III

Focus: Communication with asthma patients

1. If you would like to convince a patient who used SABA more than twice per week to cut down on the use, how would you convey this message?
2. How likely do you think that asthma patients will talk about their asthma conditions with community pharmacists?

Part IV

Focus: Future perspectives

1. What could be the possible suggestions to improve the safe use of asthma reliever inhalers which are requested from community pharmacies.
2. What could be the possible suggestions to improve the communications between patients and community pharmacists?

Conclusion/suggestion:

1. Would you like to provide any additional suggestions about what the policy makers or Ministry of Health could do to improve the safe use of asthma medicines in Malaysia?

**Appendix C.**

**Perceptions and practices of community pharmacists towards the use of short-acting beta-2 agonists (SABA) inhalers: A cross-sectional study**

(Questionnaire)

| In this section, we are interested to know about your perceptions regarding patients’ asthma control and management. Please indicate how much you agree with each of the following statements.  1= Strongly disagree, 2 = Disagree, 3 = Neutral, 4 = Agree, 5 = Strongly agree | | | | | |
| --- | --- | --- | --- | --- | --- |
| **Focus 1: Asthma control and management** | | | | | |
| 1. According to you, good asthma control is: | | | | | |
| a. Asthma that can be controlled with medical help. | ◯  1 | ◯  2 | ◯  3 | ◯  4 | ◯  5 |
| b. Having minimum asthma symptoms. | ◯  1 | ◯  2 | ◯  3 | ◯  4 | ◯  5 |
| 2. Do you perceive that it is reasonable for patients to change their asthma management if they really feel unwell on a particular day? | ◯  1 | ◯  2 | ◯  3 | ◯  4 | ◯  5 |
| 3. Beside the medicines that were prescribed by physicians, do you think that patients managed their asthma with other over the counter (OTC) medicines? | ◯  1 | ◯  2 | ◯  3 | ◯  4 | ◯  5 |
| 4. Do you perceive that patients manage their asthma by decoction, herbal, or ayurvedic medicines? | ◯  1 | ◯  2 | ◯  3 | ◯  4 | ◯  5 |
| 5. Asthma patients who always purchase SABA inhalers from community pharmacies will lead to poor asthma control due to the absence of asthma review at hospitals. | ◯  1 | ◯  2 | ◯  3 | ◯  4 | ◯  5 |
| 6. Asthma patients are willing to talk about their asthma problems to community pharmacists. | ◯  1 | ◯  2 | ◯  3 | ◯  4 | ◯  5 |

| In this part, we are interested to understand about your perceptions of risks involve in asthma relievers use. Please indicate how much you agree with each of the following statements.  1 = Strongly disagree, 2 = Disagree, 3 = Neutral, 4 = Agree, 5 = Strongly agree | | | | | |
| --- | --- | --- | --- | --- | --- |
| **Focus 2: Risk perceptions of asthma reliever inhalers** | | | | | |
| 1. Do you think that it is safe for patients to use more than 2 puffs of SABA inhalers per day? | ◯  1 | ◯  2 | ◯  3 | ◯  4 | ◯  5 |
| 2. If someone uses 2 or more puffs per day, most probably he / she is having uncontrolled asthma. | ◯  1 | ◯  2 | ◯  3 | ◯  4 | ◯  5 |
| 3. Asthma patients should move to another inhaler completely which contained both a preventer and a reliever. | ◯  1 | ◯  2 | ◯  3 | ◯  4 | ◯  5 |
| 4. Do you think that asthma patients develop attachment to SABA inhalers? | ◯  1 | ◯  2 | ◯  3 | ◯  4 | ◯  5 |
| 5. Asthma patients may experience side effects from the use of SABA inhalers.   (For examples, dry mouth, palpitations, tremor, chest tightness, muscle cramps, headache) | ◯  1 | ◯  2 | ◯  3 | ◯  4 | ◯  5 |
| 6. There is a high chance that asthma patients overuse SABA inhalers which are requested from community pharmacies compared with inhalers which are obtained from hospitals or clinics. | ◯  1 | ◯  2 | ◯  3 | ◯  4 | ◯  5 |
| 7. Are you more concerned about the frequencies and doses of asthma patients’ SABA usage compared with the techniques to utilize the inhalers? | ◯  1 | ◯  2 | ◯  3 | ◯  4 | ◯  5 |

| In this section, we are interested to know about the practices of community pharmacists towards SABA usage. Please indicate how much you agree with each of the following statements.  1 = Strongly disagree, 2 = Disagree, 3 = Neutral, 4 = Agree, 5 = Strongly agree | | | | | |
| --- | --- | --- | --- | --- | --- |
| **Focus 3: Practices towards the use of SABA inhalers** | | | | | |
| 1. Pharmacists could dispense the SABA inhalers to asthma patients if they noticed that they might overuse it. | ◯  1 | ◯  2 | ◯  3 | ◯  4 | ◯  5 |
| 2. Pharmacists could not dispense the SABA inhalers to asthma patients if they noticed that they might overuse it. | ◯  1 | ◯  2 | ◯  3 | ◯  4 | ◯  5 |
| 3. Have you ever educated the patients regarding the correct techniques to use asthma inhalers? | ◯  1 | ◯  2 | ◯  3 | ◯  4 | ◯  5 |
| 4. At my workplace, I believed that I provide enough information regarding the safe use of SABA inhalers to the patients. | ◯  1 | ◯  2 | ◯  3 | ◯  4 | ◯  5 |

| In this part, we are interested to identify the possible factors that contribute to the safe use of SABA inhalers. Please indicate how much you agree with each of the following statements.  1 = Strongly disagree, 2 = Disagree, 3 = Neutral, 4 = Agree, 5 = Strongly agree | | | | | |
| --- | --- | --- | --- | --- | --- |
| **Focus 4: Facilitators to the safe use of SABA inhalers** | | | | | |
| 1. A special room dedicated for asthma consultations. | ◯  1 | ◯  2 | ◯  3 | ◯  4 | ◯  5 |
| 2. Improve the duration of asthma counselling. | ◯  1 | ◯  2 | ◯  3 | ◯  4 | ◯  5 |
| 3. A monitoring system to keep track of the SABA inhalers purchasing records in the community setting. | ◯  1 | ◯  2 | ◯  3 | ◯  4 | ◯  5 |
| 4. Provide more follow up sections to the asthma patients. | ◯  1 | ◯  2 | ◯  3 | ◯  4 | ◯  5 |

| In this part, we are interested to examine the possible factors that could hinder the safe use of SABA inhalers. Please indicate how much you agree with each of the following statements.  1 = Strongly disagree, 2 = Disagree, 3 = Neutral, 4 = Agree, 5 = Strongly agree | | | | | |
| --- | --- | --- | --- | --- | --- |
| **Focus 5: Barriers to the safe use of SABA inhalers** | | | | | |
| 1. Lack of time. | ◯  1 | ◯  2 | ◯  3 | ◯  4 | ◯  5 |
| 2. Increased workload. | ◯  1 | ◯  2 | ◯  3 | ◯  4 | ◯  5 |
| 3. Less / no incentives. | ◯  1 | ◯  2 | ◯  3 | ◯  4 | ◯  5 |
| 4. Lack of training. | ◯  1 | ◯  2 | ◯  3 | ◯  4 | ◯  5 |
| 5. Lack of patient awareness of the services. | ◯  1 | ◯  2 | ◯  3 | ◯  4 | ◯  5 |
